# Editorial Department

**Published:** 1847-05

**Authors:** 


In the present state of the question relative to the priority of discovery of the application of Sulphuric Ether to surgical operations, we deem the following of sufficient importance to be copied entire.

"It has been repeatedly said that Dr. Jackson is not concerned in the Patent for the Letheon ; that Mr. Morton alone has taken out the Letters Patent, and that whatever interest Dr. J. may have in it, arises out of some private contract between them. But it now appears that Dr. J. is really one of the proprietors—that the patent is issued in favor of Jackson and Morton conjointly.

"The question has been asked, probably by every member of the profession, "what is patented ?" I put the question, the other day, to a gentleman in Boston, who ought to know, and he replied, "The inhalation of the letheon by means of a valvular apparatus." This answer is far from satisfactory, for the same effect may be produced by inhalation of the vapor, without any valvular apparatus at all. If the "apparatus" is an essential part of the patent, the use of a different apparatus would enable any one to evade the penalty of the law. Have they patented the production of insensibility to pain by the inhalation of etheric vapor ? No. A physician may administer the vapor, and produce insensibility to pain, with or without the valvular apparatus, without infringing upon the patent. He may administer it for the headache, the heartache or the bellyache, for tic douloureux, asthma or hysterics, and the patent will not reach him. Indeed, I am not quite sure that the patent will reach him if he uses the vapor in reducing dislocations or hernia, or in any other operation in which "the knife or other instrument of operation of a surgeon" is not used.

What, then, is the precise thing patented? I answer, the combining with surgical operations the application of ether, or the vapor thereof. This is the whole thing. The use of it in the practice of medicine, by inhalation, is not patented, nor in surgery even, except when connected with operations. They claim the right to use an old and well-known medicine to produce a given result, in the treatment of certain cases. The principle, then, is that a member of the profession, if he discover that a certain effect may be produced by any remedy or agent in common use, when used in a specified manner, in a certain case or class of cases, which effect had not (to his knowledge) been previously produced by said remedy or agent, he may secure to himself, by patent, the use of said article for producing this specified effect. For instance, should I discover that tinct. digitalis would cure Dixon Lewis, and others similarly affected, of excessive obesity, as it probably would, I might patent the use of tinct. dig. in such cases. If I discover that hydriod. potassæ, applied in a particular way, will cure dry scab, or scurfy eruptions of the skin and scalp, I may patent this particular use of it, in this class of cases, and require my bretheren to pay me a stipulated sum, or a certain per cent. of the fees they may receive, for the right to use it in such cases. Should I discover that tinct. cayenne pepper and tinct. opii, combined in certain proportions, will cure the cholera, I may claim the sole right to use them in cholera, however many persons may be dying around me for the want of them. If some *Yankee* were now in Badgad, with a few gallons of these tinctures, with their use secured to him by patent, would not he coin money?

The use of a known remedy to produce a particular effect in any given branch of professional practice, or in the treatment of a given class of cases, is the principle involved. This, so far as I can discover, from a careful examination of the specification, is the exact principle implied in it. As to the rectitude of this principle, professionally, socially or morally, I say nothing. Each one can judge for himself. I believe the illustrations I have used above are correct and appropriate—that is, if surgery and the practice of medicine are parts of one and the same profession. If surgery is a mere mechanical operation, and is to take its place in the same category as other operations in mechanics, then the case is altered. Success in the mechanic arts, depends, not only upon the skill with which their process is accomplished, but often upon the processes themselves, and when a man invents a process by which the same result can be accomplished better than before, he is permitted, by common consent, to enjoy the benefit of his invention for a limited time. If surgery puts in the same claim for its inventions, let it be divorced from the liberal professions—from the "humanities," and hang out before its office doors, as in the days of Ben Johnson, a staff wound with a red tape, as a *sign* that "surgery is done here." We all know the origin of the barber's pole ; and, Mr. Editor, there is a more close connection between surgery and barbery, than one would at first imagine. Many of the operations of *surgery* are *barbarous*, and the operations of *barbery* are often surgical. Indeed, many a poor wight would consider it no small alleviation of one of the miseries of human life, could he inhale the letheon before submitting to the most common operations of barbery. Mem. Barbers may use the letheon without infringing upon the patent. With the above remarks, which have extended much farther than I intended, I send you a copy of the specification, which has recently come into my hands, thinking it will gratify the curiosity of many of your brethren. Yours, S.

*March*, 1847.

"*The United States Patent Office.*—To all persons to whom these presents shall come greeting : This is to certify, that the annexed is a true copy upon the record of this office, of the specification of Jackson and Morton's Letters Patent, dated 12th Nov., 1846.

"In testimony whereof, I, Edmund Burke, Commissioner of Patents, have caused the seal of the Patent office to be hereunto affixed, this 12th day of February, in the year of our Lord one thousand eight hundred and forty-seven, and of the Independence of the United States the seventy-first. EDMUND BURKE."

*The Schedule referred to in these Letters Patent, and making part of the same.*

To all persons to whom these presents shall come : Be it known, that we, Charles T. Jackson and William T. G. Morton, of Boston, in the County of Suffolk and State of Massachusetts, have invented or discovered a new and useful improvement in surgical operations on animals, whereby we are enabled to accomplish many, if not all operations, such as are usually attended with more or less pain and suffering, without any or very little pain to, or muscular action of persons who undergo the same ; and we do hereby declare that the following is a full and exact description of our said invention or discovery.

It is well known to chemists that when alcohol is submitted to distillation with certain acids, peculiar compounds, termed *ethers*, are formed ; each of which is easily distinguished by the name of the acid employed in its preparation. It has also been known that the vapors of some, if not all these chemical distillations, particularly those of sulphuric ether, when breathed or introduced into the lungs of an animal, have produced a peculiar effect on its nervous system; one which has been supposed to be analogous to what is usually termed intoxication. It has never (to our knowledge) been known until our discovery, that the inhalation of such vapors (particularly those of sulphuric ether) would produce insensibility to pain, or such a state of quiet of nervous action as to render a person or animal incapable to a great extent, if not entirely, of experiencing pain while under the action of the knife, or other instrument of operation of a surgeon, calculated to produce pain. This is our discovery, and the combining it with or applying it to any operation of surgery, for the purpose of alleviating animal suffering, as well as of enabling a surgeon to conduct his operations with little or no struggling or muscular action of the patient, and with more certanty of success, constitutes our invention. * * *

In order to render the ether agreeable to various persons, we often combine it with one or more essential oils, having pleasant perfumes. This may be effected by mixing the ether and essential oil, and washing the mixture in water. The impurities will subside, and the ether, impregnated with the perfume, will rise to the top of the water. We sometimes combine a narcotic preparation, such as opium or morphine, with the ether. This may be done by many ways known to chemists, by which a combination of etheric and narcotic vapors may be produced. * * * * *

From the experiments we have made we are led to prefer the vapors of sulphuric ether to those of muriatic or other kinds of ether, but any such may be employed which will properly produce the state of insensibility without any injurious consequences to the patient.

We are fully aware that narcotics have been administered to patients undergoing surgical operations, and, as we believe, always by introducing them into the *stomach*. This we consider in no respect to embody our invention, as we operate through the *lungs and air passages*, and the effects produced upon the patient are entirely or so far different as to render the one of very little, while the other is of immense utility. The consequences of the change are very considerable, as an immense amount of human or animal suffering can be prevented by the application of our discovery.

What we claim as our invention is the herein before described means by which we are enabled to effect the above highly important improvement in surgical operations, viz., by combining therewith the application of ether or the vapor thereof substantially as above specified.

In testimony whereof, we have hereunto set our signatures this twenty-seventh day of October, A. D. 1846.

Witnesses, CHARLES T. JACKSON,

*R. H. Eddy, W. H. Leighton.* WM. T. G. MORTON.

